# Cost-Effective Mapping of Benthic Habitats in Inland Reservoirs through Split-Beam Sonar, Indicator Kriging, and Historical Geologic Data

**DOI:** 10.1371/journal.pone.0095940

**Published:** 2014-04-23

**Authors:** Erik R. Venteris, Cassandra J. May

**Affiliations:** 1 Ohio Department of Natural Resources, Division of Geological Survey, Columbus, Ohio, United States of America; 2 The Ohio State University, Department of Evolution, Ecology, and Organismal Biology, Aquatic Ecology Laboratory, Columbus, Ohio, United States of America; University of Waikato (National Institute of Water and Atmospheric Research), New Zealand

## Abstract

Because bottom substrate composition is an important control on the temporal and spatial location of the aquatic community, accurate maps of benthic habitats of inland lakes and reservoirs provide valuable information to managers, recreational users, and scientists. Therefore, we collected vertical, split-beam sonar data (roughness [E1], hardness [E2], and bathymetry) and sediment samples to make such maps. Statistical calibration between sonar parameters and sediment classes was problematic because the E1:E2 ratios for soft (muck and clay) sediments overlapped a lower and narrower range for hard (gravel) substrates. Thus, we used indicator kriging (IK) to map the probability that unsampled locations did not contain coarse sediments. To overcome the calibration issue we tested proxies for the natural processes and anthropogenic history of the reservoir as potential predictive variables. Of these, a geologic map proved to be the most useful. The central alluvial valley and mudflats contained mainly muck and organic-rich clays. The surrounding glacial till and shale bedrock uplands contained mainly poorly sorted gravels. Anomalies in the sonar data suggested that the organic-rich sediments also contained trapped gases, presenting additional interpretive issues for the mapping. We extended the capability of inexpensive split-beam sonar units through the incorporation of historic geologic maps and other records as well as validation with dredge samples. Through the integration of information from multiple data sets, were able to objectively identify bottom substrate and provide reservoir users with an accurate map of available benthic habitat.

## Introduction

The construction of dams and reservoirs to retain and control water extends back to the ancient Egyptians and Mesopotamians [1]. Reservoirs provide water supply, flood control, navigation, and recreation opportunities. However, reservoir managers face unique challenges to maintain these benefits, the most common being the infilling of reservoirs over time due to sediment loads from tributaries [2], [3]. Understanding and modeling the controls on infilling rates [4] is important to predict the useful life of the reservoir and to plan future dredging [5], [6] and sediment flushing operations [7]. The issue of reservoir infilling is further complicated because accumulated sediments often exhibit high levels of metals [8] and other potentially toxic substances [9], [10], [11]. In contrast, the role of these bottom sediments as an ecological habitat within the reservoir ecosystem has received less attention, especially from the earth science community. A few studies have considered mapping bottom sediments in reservoirs as a benthic habitat for organisms ranging from bacteria [12] to macro-vertebrates (i.e., fish; [13]). Ecologically focused mapping of bottom sediments is essential, as they have an important role in the food web dynamics of these water bodies [14]. For example, detrital organic matter supplied by the surrounding landscape is an important energy source for critical fish species, such as gizzard shad *Dorosoma cepedianum* [15]. Further, many macroinvertebrates (ex. mayfly *Hexagenia* [16]; amphipod *Pontoporeia affinis* [17]; chironimid midges [18]) and fish species (ex. walleye *Sander vitreus* [19]; black crappie *Pomoxis nigromaculatus* [20]; smallmouth bass *Micropterus dolomieu* [21]) will show preferences for specific benthic substrates in lakes or reservoirs.

Mapping of bottom sediments in marine and freshwater environments is commonly based on sonar data and backscatter analysis [22] (e.g., multibeam and side scan), calibrated using sediment samples. Reservoir systems offer the advantage of being much younger than natural systems such as marine and lacustrine environments, and therefore pre-flood geological and other historical data are often available and can be used to inform the process of sediment mapping. Consequently, we propose that backscatter analysis can be made more accurate, efficient, and provide more insight into sediment types when data representing the complex natural history of the reservoir are considered.

For Hoover Reservoir and other flooded landscapes, geomorphic hill slope processes and drainage development shaped the original pre-flood basin. The underlying geologic materials (bedrock and sediments) influenced the topography of the valley and served as the parent material for soils and sediment. Past land use and land cover may also be important as bottom targets—building foundations, tree stumps, and roads—may remain and confuse interpretations. In addition, large amounts of soil and rock are typically removed and emplaced for construction of the dam, causeways, and other structures. Finally, sedimentary processes (e.g., wave wash, sediment from inflowing streams, mass wasting of unstable banks, etc.) actively redistribute geologic and organic materials throughout the reservoir. All of the above factors have complex interactions, which ultimately relate back to the shape of the basin and its base geologic materials. The relative importance of these processes is likely unique to each reservoir, depending on the overall environmental setting and history.

In response to this complexity, we tested a Geographic Information System (GIS)-based approach to data compilation and mapping. Previous bottom-typing studies based on vertical sonar concentrated on large, open-water applications and techniques focused on establishing statistical correlations between the acoustic measurements and sediment samples (e.g., [23]). In this work spatial models are not only based on such correlations, but also on relationships with other spatial data (proxies for the above-described processes). A goal of our work was to illustrate a basic objective methodological approach to apply to other reservoir or lake mapping projects, as specific data sets important for mapping this reservoir may not apply to others.

## Study Site

**Figure 1:**
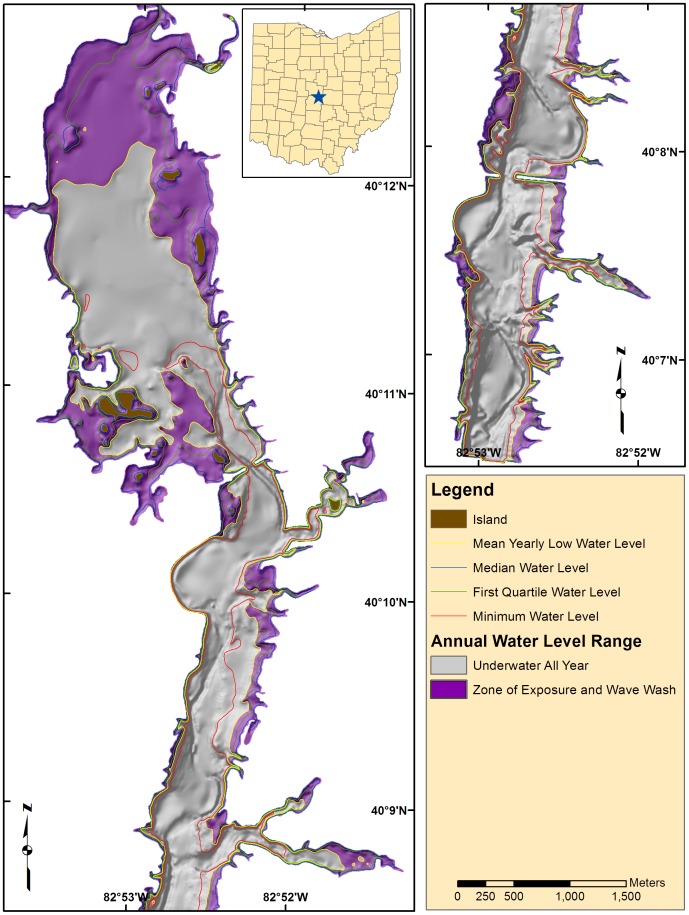
A map showing the bathymetric shaded relief of Hoover Reservoir, in Columbus, Ohio, with statistical water levels delineated, based on monthly records from 1956 to 2006. The inset map shows the location of the reservoir within the state of Ohio.

The study site was Hoover Reservoir (Fig. 1; lat 40°9′8′′N., long 82°52′36′′W.), an impoundment completed in 1955 to supply water for the city of Columbus, Ohio, that also is used extensively for recreation. The land cover of the flooded area was rural in nature, containing agricultural fields, roads, and forest. The source water for the reservoir is Big Walnut Creek, which flows north-to-south through a largely agricultural watershed (although urbanization is ongoing). Hoover Reservoir at full pool is approximately 1,176 ha in area and has a maximum depth of 18.3 m (Fig. 1). No specific permissions were required for our research and our field study did not involve endangered or protected species.

## Materials and Methods

The sediment mapping methodology in this contribution emphasizes ecological applications and results were used to specifically target the needs of fisheries managers [24]. Due to the ecological emphasis, bottom-sediment type categories (collected with a small dredge) were based on the Qualitative Habitat Evaluation Index (QHEI; [25]), rather than traditional, geologic texture categorization schemes (e.g., [26]). This basic approach to defining texture classes was considered sufficient for biological applications and was compatible with the sonar system's capability to resolve sedimentary textures.

### Sonar Data

**Figure 2:**
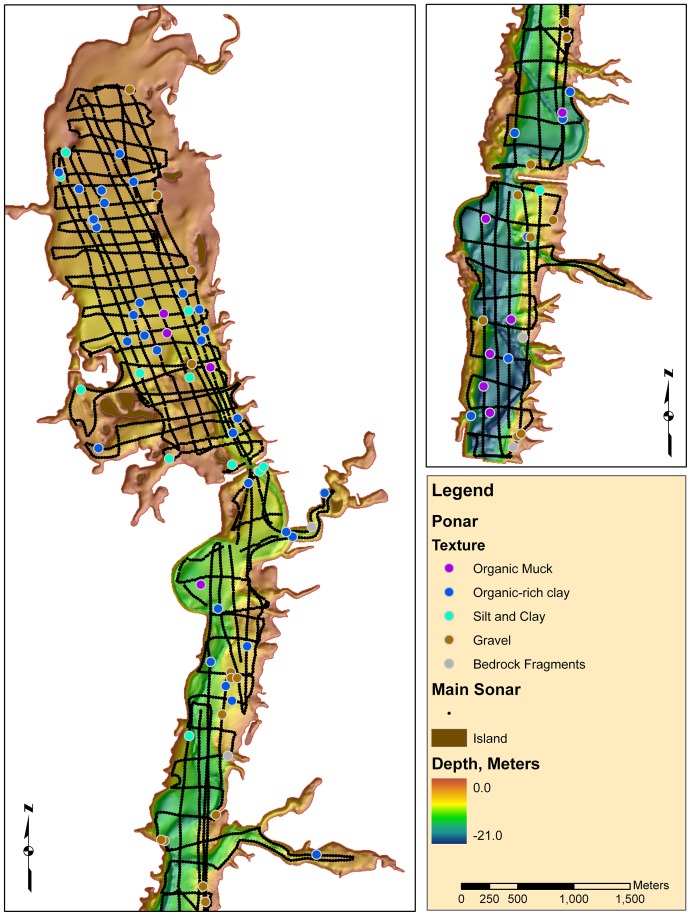
A map showing sonar data points, dredge sample calibration data points (with the sediment type indicated) and bathymetry of Hoover Reservoir in Columbus, Ohio.

We collected sonar data using a BioSonics DT-X series echosounder, which was mounted vertically to a flat-bottom, aluminum boat. The instrument's transducer had a 200-kilohertz (kHz) split-beam with a 7-degree circular cone shape. The transducer settings included pulse width of 0.4 millisecond at 10 pings/second, −70 decibels (db) threshold, and a start range of 1 m and a stop range from 10 to 20 m depending on the local shape of the reservoir. The transducer was calibrated using a tungsten carbide reference sphere following procedures outlined in [27]. Each sonar data point represents the average of 20 pings, giving a spatial resolution of approximately 20 m [28]. We collected the data along a series of north–south and east–west transects in the spring of 2006. There were 5,039 individual measurements of depth, roughness, and hardness (Fig. 2).

The sonar data were post-processed using Echoview 3.1 software (from Myriax Software) to obtain water depths and backscatter measurements of bottom roughness (tail of first echo) and hardness (second echo) ([29],[23]; similar to the RoxAnn method of [30]). Roughness was measured from the integral of the portion of the first echo that occurs after the returns from the initial incident angles (the initial returns contained strong, undesirable amplitude contributions from bottom reverberations). Hardness was defined as the integral of the second echo. The ratio of roughness (E1 for first echo) to hardness (E2 for second echo), hereafter referred to as E1:E2, was also used for bottom-sediment typing as an alternative to using plots of E1 versus E2 to identify clusters related to sediment type [23].

### Ponar Calibration Data

We collected calibration data to establish relationships between sonar data and bottom sediment type. Sediment samples and sonar data were collected at 88 locations using a Wildco Petite Ponar Grab (dimensions 152 mm×152 mm). We located sample sites that encompassed a range of roughness and hardness combinations. Sediments were recovered and described in the field based on observation and hand texturing. The descriptions were summarized into five texture categories (Table 1, Fig. 2).

**Table 1:** Sediment descriptions from the ponar samples at Hoover Reservoir in Columbus, Ohio.^a^

Texture Class	QHEI^b^ Substrate	N	Hydroacoustic Facies Class	Description
Muck	Muck	10	1	Dark, low bulk density, composed primary of decomposed organic matter
Organic-Rich Clay	Hardpan^c^	39	1	Dark, clay sediment with organic matter; higher bulk density than muck
Silt and Clay	Silt	13	0	Fine-grained sediments lacking significant organic matter content
Gravel	Gravel	22	0	Poorly sorted sediment composed of gravel, sand and silt; gravel fragments rounded, if derived from till, or angular fragments of shale
Bedrock Fragments	Gravel^d^	4	0	Composed primarily of shale fragments, with minor amounts of finer-grained sediment

a The 88 total samples were put into five categories, chosen to best describe the sediments encountered.

b QHEI equivalent units are given, but some incompatibilities were noted as QHEI is best applied to streams (as described below).

c The organic-rich clays are not dense and hard as described in the QHEI but fit most closely into the hardpan category.

d The bedrock fragments are gravel in size but are not rounded as in the formal QHEI category.

### Supporting Spatial Data

When creating maps by statistical interpolation of point samples, it is often advantageous to incorporate additional spatial data. Even if not quantitatively included in the model, such data can be used to aid interpretation and better understand anomalies. The central idea is to find spatially continuous data sets that are statistically correlated or otherwise related to property to be mapped or modeled. For our study, GIS data sets containing information on bathymetry, sedimentation, pre-flood land cover, water level fluctuations, and geology were investigated as potential environmental correlates.

The Ohio Department of Natural Resources (ODNR), Division of Wildlife created a bathymetric map [31] (Fig. 2) based on sonar soundings collected in 2001 using a combination of computer interpolation (kriging) and manual digital contouring. Manual corrections were needed near-shore where the sonar data could not be collected because of water depth limitations. The bathymetric contours were converted to grids for display and GIS analysis using Topogridtool in ESRI ArcGIS.

We used the bathymetric map in conjunction with water level data (provided by the City of Columbus, Division of Water) to map annual fluctuations in the position of the waterline (Fig. 1). The average depth of the reservoir is 6.6 m at the full pool elevation of 272.6 m above sea level (median pool is 271 m). The reservoir water level fluctuates annually, reaching maximum levels in April and minimum levels in November. The average annual water level fluctuation (max–min) is 3.7 m, mainly due to annual drawdown.

**Figure 3:**
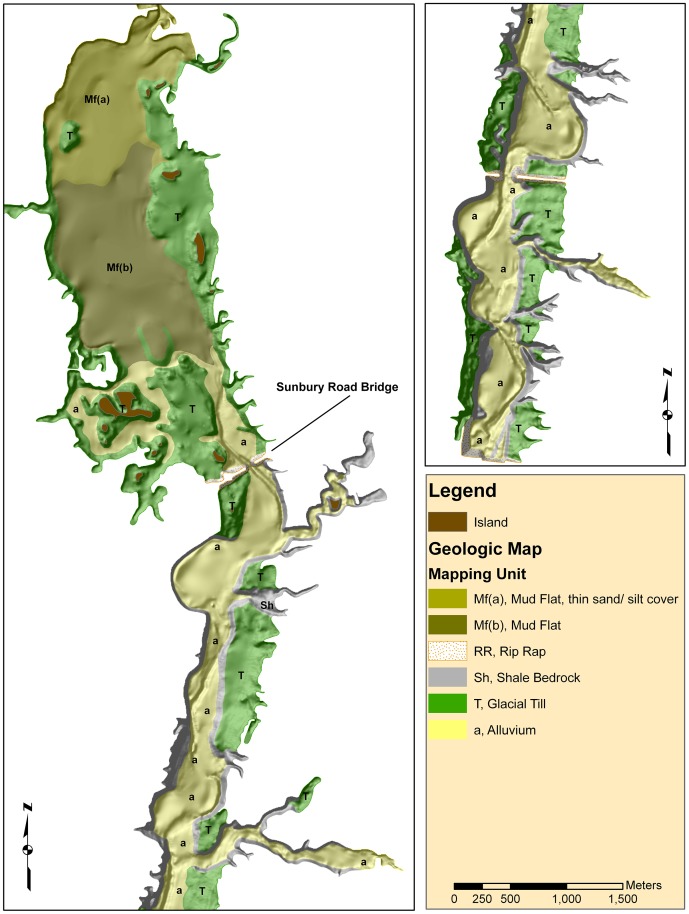
A map showing the generalized near surface geology of Hoover Reservoir in Columbus, Ohio. The map was drawn by interpreting the landforms apparent in the bathymetry contoured from sonar data and field investigations. The mapping units are described in Table 1.

We created a general geologic map of surficial materials (polygons defining areas of unconsolidated sediment or bedrock types (where exposed as outcrops) within one meter of the surface)). Mapping was partly based on field work conducted in autumn 2008, when the reservoir was drawn down to an unusually low level. The boundaries of the geologic map were delineated on the basis of geomorphic interpretation of landforms apparent in the field and in the bathymetric map. Interpretations were confirmed by field investigation as much as possible. In all, five individual geologic units were identified (Table 2) and mapped (Fig. 3).

**Table 2:** Geologic mapping units (Fig. 5), map symbols, and unit descriptions for the bottom sediments at Hoover Reservoir in Columbus, Ohio.

Map Symbol	Name	Description, Primary Material
Mf(a)	Mud flat, sand covered	Holocene-age, mud flat with thin covering of fine sand and silt; surface texture grades from fine sand at the periphery (near shore) to clay near the center of the unit
Mf(b)	Mud flat	Holocene-age, mud flat containing organic-rich clay sediments
Sh	Shale Bedrock	Devonian-age, fissile shales interbedded with occasional siltstones
T	Glacial Till	Wisconsin-age, glacial till containing igneous and limestone boulder clasts and shale fragments in a fine, silty clay matrix
a	Alluvium	Holocene-age, alluvial silts and sands associated with the pre-reservoir floodplain; not exposed during the 2008 field work and was interpreted from geomorphic landforms
RR	Rip Rap	Limestone boulders emplaced for the construction of dams, causeways, and shore protection

**Figure 4:**
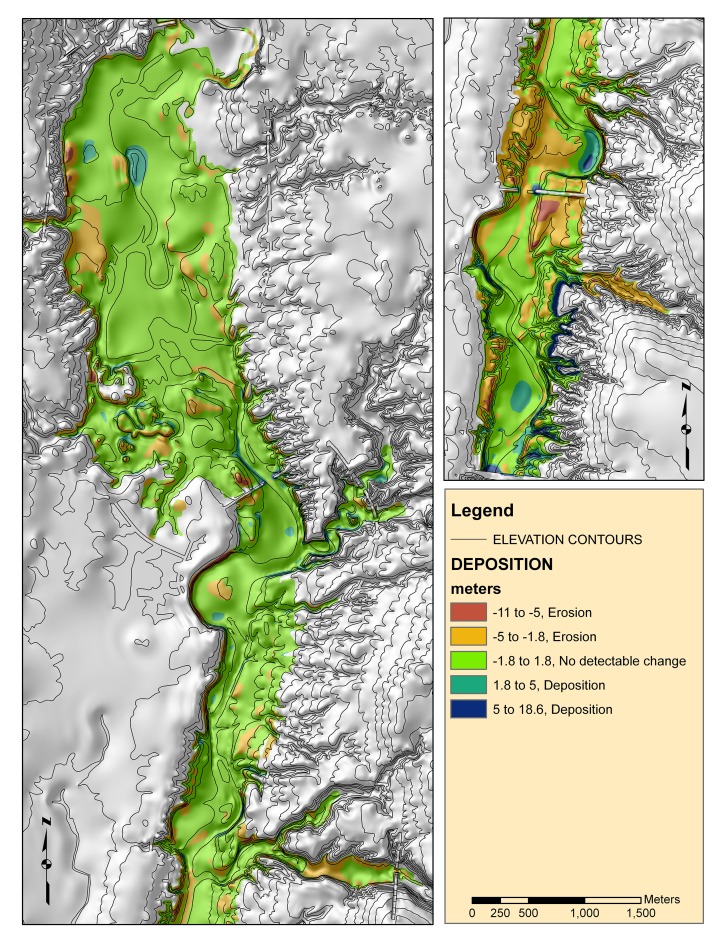
Map showing elevation contours from the United States Geological Survey and the difference between the bathymetry from sonar and the bathymetry from the pre-flood topography map of Hoover Reservoir.

**Figure 5:**
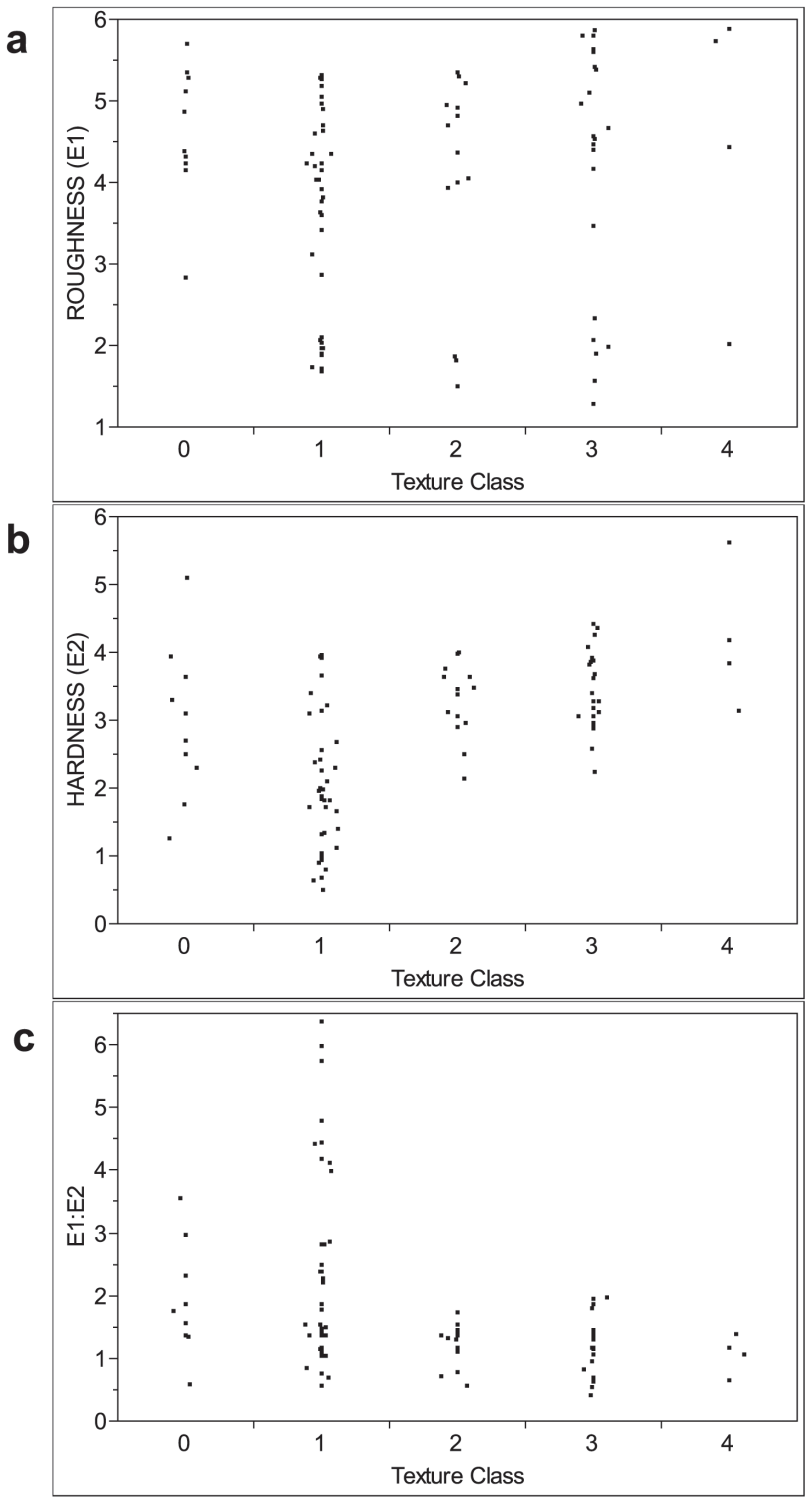
Figure showing scatter plots of (a) roughness, (b) hardness, and (c) E1:E2 ratio by the bottom sediment classes of the calibration data (Table 1) for Hoover Reservoir.

The reservoir is divided into two main geomorphic units. The upper portion (the mudflat mapping units, Mf(a) and Mf(b)) is a wide (1.0 km), relatively shallow valley with gradual slopes. Pre-flood topography (Fig. 4) is similar to the current bathymetry, so the topography is not the result of post-flood sedimentation. Rather the till is thick (∼30 m) and bedrock is not exposed or near the surface, so lateral erosion was not constrained by bedrock. This contrasts with the lower portion (south of the Sunbury Road bridge, middle of the left panel in Fig. 5) where the bedrock base of the valley is narrow (0.5 km), the till is thin (pinching out towards the valley center), and outcrops of shale bedrock are prevalent. Here, the valley floor contains a distinct, relatively flat floodplain filled with alluvial (silty) sediments.

Historical GIS data was also collected to aid in interpretation. Elevation contours from the pre-flood (pre-1955) surface were available at the 1∶24,000 scale from the United States Geological Survey (USGS). These contours were converted to a grid for display and GIS analysis using Topogridtool in ArcGIS (ESRI, 2008). The original USGS contours, a shaded-relief model, and a grid of the difference between the sonar-derived map and the elevation contours (similar to a study done by [3]) are shown (Fig. 4). The map shows net erosion or deposition over the time period 1955–2001. An aerial-photo mosaic from the USGS was also located and georeferenced to provide information on land cover features within the reservoir before flooding (1953).

### Mapping Methods

We used geostatistical interpolation [32] to create an initial map to better visualize the spatial patterns in the sonar data and to compare these patterns with potential spatial correlates. It was important to consider the E1:E2 distribution when choosing the interpolation method, as it was highly skewed and contained extreme values. While ordinary kriging (OK) does not require a specific distribution form, extreme values cause the assumption of linear weighting independent of data values to be suspect. To avoid these potential issues, geostatistical mapping was based on a binary transform of the data, through a technique known as indicator kriging (IK) [33]. Formally, IK provides a least-squares estimate of the conditional cumulative distribution function 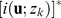, for the binary indicator variable *i* at location **u** for a specified cutoff *z_k_*

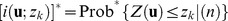
(1)where *Z* is a random variable at location **u**, and *n* is the local conditioning information (data values and their distance weighting determined by kriging) [32]. A threshold value of *z_k_* was used to transform the continuous sonar data to the binary indicator variable (*i*) according to
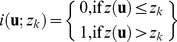
(2)


The value of *z_k_* can be chosen on many bases; here the choice was based on the results of data exploration. The local conditioning information (*n*) was estimated for the transformed data using OK, as implemented in Geostatistical Analyst, an extension within ArcGIS software (ESRI, 2008).

We chose the IK model parameters using the following procedure. The calibration data showed that a value of 2.00 in the E1:E2 ratio was not exceeded for the silt and clay, gravel, and bedrock fragment texture classes (Fig. 5). Therefore, *z_k_* was set to 2.00 for IK, with areas exceeding this E1:E2 ratio unlikely to contain hard substrates. We calculated an experimental variogram using a lag spacing of 50 m over 10 lag intervals. A model semi-variogram was fit using an isotropic, spherical variogram with a range of 370.8 m, a partial sill of 0.16 and a nugget effect of 0.075. The points used for calculating the kriging estimate were selected using four quadrants rotated 45 degrees from north to minimize local selection bias due to the orientation of the transect lines. Up to six points were selected from each quadrant for a maximum of 24. Only points within the autocorrelation range (370.8 m) were selected for estimation of kriging weights.

In addition to mapping by kriging, we used traditional, non-spatial statistical methods (e.g., ANOVA). All statistical analyses utilized JMP software (from SAS Institute Inc.). We explored the relationships between the sonar data, calibration data and the GIS data sets collected in order to derive a final, scientifically justifiable, map of bottom sediments.

## Results

### Exploratory Data Analysis

We conducted exploratory data analysis to detect potential outliers and determine appropriate statistical methods. Two data sets were considered—the full mapping sonar (*n* = 5,039 samples) and the sonar with dredge samples for calibration (*n* = 88 samples). The data distributions for the full mapping data set were not Gaussian normal. The roughness distribution had long tails; hardness showed a bimodal distribution and the E1:E2 ratio was heavily skewed towards small values.

We sought statistical calibration models between sediment type (dredge samples) and the sonar indices. As a first step, the distributions in roughness, hardness, and E1:E2 between the basic sediment types were investigated (Fig. 5). Roughness alone contained little useful information, as the values overlapped and all sediment types except muck showed a bimodal distribution. The muck and organic-rich clay classes had the largest range in both hardness and E1:E2 values, whereas the remaining texture classes had a more restricted range, generally larger hardness and smaller E1:E2 values. The E1:E2 ratio provided the clearest information on sediment type (Fig. 5c), showing two distinct hydroacoustic sediment types (Table 1). The sediments containing appreciable organic matter covered a wide range of E1:E2 values, but the coarser fractions did not exceed an E1:E2 ratio of 2.00. Hence, there was information on where coarse, hard sediments did not occur, but there was a zone of overlap where all sediment classes were represented. The overlap was the main challenge to this case study and demonstrates that sonar-based hardness and roughness measurements may have limited function as “bottom sediment detectors” without further data to support interpretation and modeling.

### Mapping

**Figure 6:**
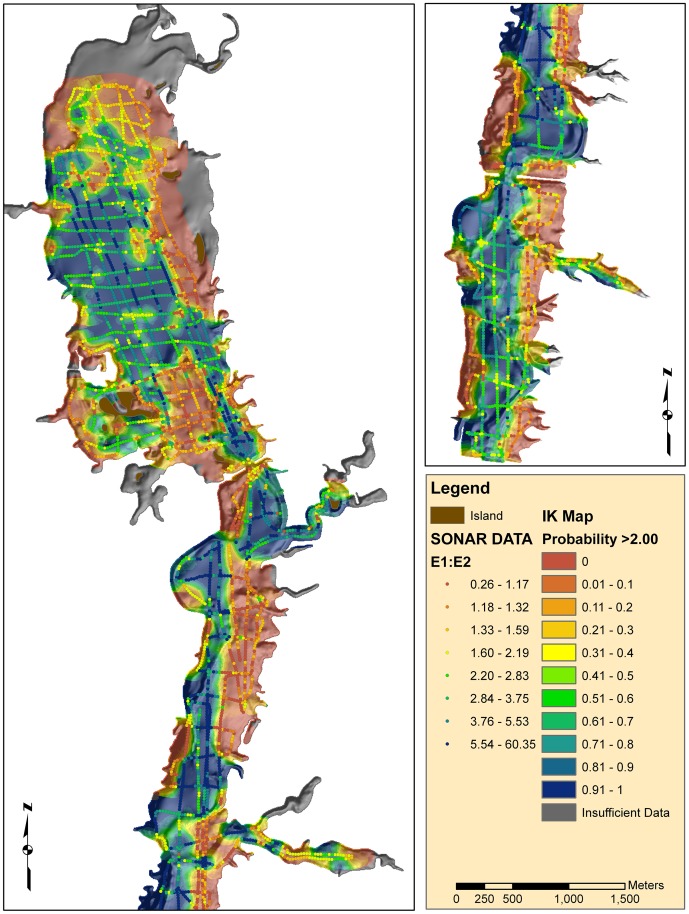
Map showing indicator kriging results, values are the probability of exceeding an E1:E2 (roughness:hardness) ratio of 2.00. From the calibration data, the coarse sediments (hydrofacies 0; see Table 1) do not have E1:E2 ratios exceeding 2.0.

Geostatistical modeling was used as a straightforward method to visualize the sonar data and generate appropriate hypotheses for further testing. The IK mapping results (Fig. 6) show distinct spatial patterns, with the central portion of the reservoir unlikely to contain hard bottom sediment types as would be expected from sedimentation patterns. At question was the best method to convert the geostatistical map into a categorical one to clearly communicate the spatial distribution of the most likely bottom sediment types to a range of stakeholders. The overlap in sonar values between the sediment categories precluded direct calibration and conversion. Additional predictive spatial data were explored in order to better resolve this ambiguity.

Several spatial data sets were compared to the data and IK map to test as predictive variables. One hypothesis was that the hard bottom areas were related to lag deposits left behind by wave action. Therefore, the pattern of hardness and roughness would correlate to the average annual zone of wave wash. Contrarily, small E1:E2 values (Fig. 6) occur well below the average annual low stand (Fig. 1). The patterns do show similarities in the upper basin, (border between Mf(a) and Mf(b)), but overall the wave wash zone was not consistent with the E1:E2 spatial patterns. We investigated construction effects and sedimentation from the map of erosion and deposition. There were areas with large differences (Fig. 4) at basin centers and near construction features (the dam and causeways) that exceeded the uncertainty associated with the elevation models (∼1.8 m RMSE, based on propagation of DEM and bathymetry errors). While it is likely that sedimentation is important to bottom type, most of the reservoir showed elevation differences that were below detectable limits (Fig. 4). Another hypothesis was that sonar patterns might be related to bottom targets such as trees and foundations left over from the pre-flood landscape. Visual comparisons between the sonar data and archived aerial photography showed little correlation between past land cover type and sonar measurements.

The spatial patterns in the sonar measurements were closely related to landform and surficial geology. The IK map (Fig. 6) produced spatial patterns with a clear relationship to the geomorphologic landforms apparent in the reservoir basin (Fig. 4). In general, the highest probability of hydroacoustic facies 1 was towards the central, low-elevation portion of the reservoir, and the lowest probability was towards the peripheral uplands. Accordingly, we hypothesized that geologic mapping units Mf(a), Mf(b) and a would contain more organic and organic-rich clay sediments than the upland units (Sh, T). We tested our hypothesis using both the main sonar and calibration data sets.

We observed clear differences in sonar response between the geologic map units (Table 3). As expected, alluvial and mudflat mapping units had lower hardness values than the till and bedrock upland areas. We tested the statistical difference in mean sonar response between geologic units using Tukey-Kramer Honestly Significant Difference (HSD). The natural log of E1:E2 was used for the test, as its distribution was approximately normal. The test shows that each geologic unit—other than rip rap, which had limited samples and little coverage area—had a distinct sonar signature. The overall ordering of E1:E2 and the hardness data were consistent with expectations for the dominant geologic materials of the mapping units.

**Table 3:** Summary statistics of sonar and ponar results for each geologic unit at Hoover Reservoir in Columbus, Ohio.^a^

Map Symbol	N	Median Hardness	Mean ln(E1:E2 /T-K group	Muck n/%	Organic-rich clay n/%	Silt and Clay n/%	Gravel n/%	Rock Fragments n/%
a	1811	1.11	1.32/A	6/25	10/41.7	7/29.2	1/4.2	0/0
Mf(b)	1005	1.61	1.07/B	1/5	18/90	1/5	0/0	0/0
Mf(a)	396	2.46	0.64/C	0/50	2/50	1/25	1/25	0/0
RR	17	2.55	0.63/CD	-	-	-	-	-
Sh	498	3.11	0.47/D	2/11.1	5/27.8	1/5.6	8/44.4	2/11.2
T	1312	3.51	0.24/E	1/4.6	4/18.2	3/13.6	12/54.6	2/9.0

a Units are arranged in order of increasing bottom sediment hardness. Columns *N*, *Median Hardness*, and *Mean ln (E1:E2)/T-K group* refer to the full mapping sonar data set; the remaining columns refer to the ponar data set. A significance level of 0.05 was used for the Tukey-Kramer test.

The calibration data did not have enough samples for each combination of sediment and mapping unit to support rigorous statistical tests, but results are consistent with the sonar measurements (Table 3). Mapping units a and Mf(b) had low hardness values and were mainly composed of organic and clay-rich sediments (79.5 percent acoustic facies 1). In addition, the silt and clay category was considered “soft” as the percentage of non-gravel substrate was 97.7 percent for these units. Mapping unit Mf(a) had moderate hardness and a range of sediment types. The amount of calibration data was small for this unit, but it was well exposed during low-water levels for field investigation. The unit was underlain by soft clay sediments but has a range of surface sediment textures, ranging from fine sands near the shoreline that transitioned from silt to clay towards the deeper water areas, with muck being generally absent. The upland units (T and Sh) had large hardness values and were mainly composed of gravel sized rock fragments (55.6 percent for Sh and 63.6 percent for T). However, fine-grained sediment samples were also common for these units, typically occurring as thin (<10 cm) deposits in local depressions. The sediment sample results for the shale bedrock unit showed an unexpected range of sediment types. Field observations showed that steep areas exposed during the drawdown were clearly bedrock. However, steep areas at greater depths appear to have a mantle of soft sediment as sediment samples were not all shale nor were all sonar returns indicative of hard substrate. Such steep and deep areas were included in geologic mapping unit a.

**Figure 7:**
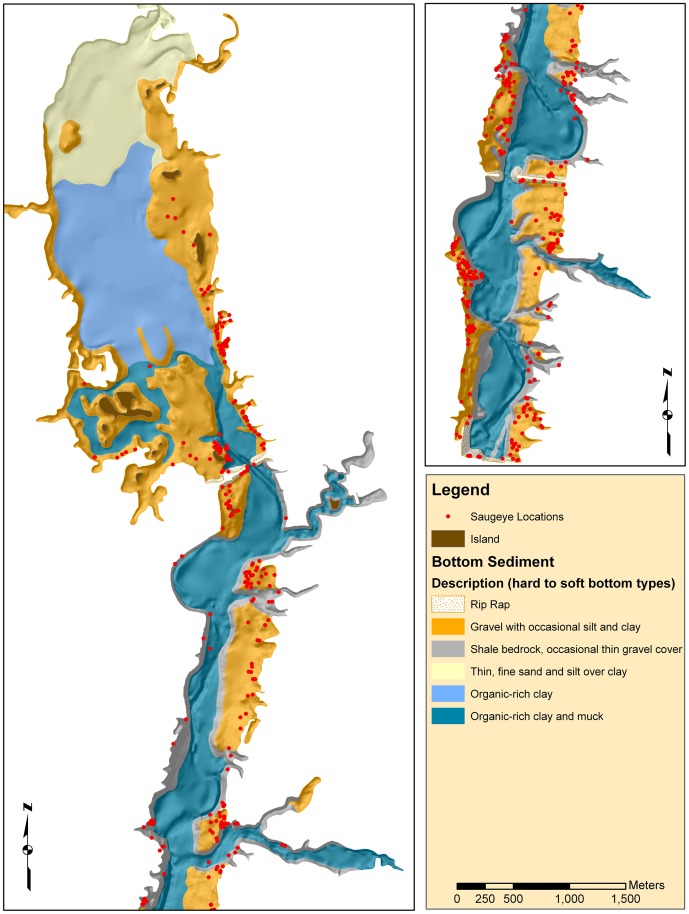
Map showing the final assignment of sediment bottom type superimposed on a bathymetric shaded relief map of Hoover Reservoir. Summary descriptions are based on data contained in Table 3. Red dots represent saugeye locations recorded from telemetry [24].

We used the results above to make a final map of bottom sediments (Fig. 7). The mapping units were based on the geologic map polygons but were assigned bottom type descriptions that best summarized the results described above. Units in the legend are arranged by relative hardness, and the main bottom sediment types are described for each unit.

## Discussion

Our goal was to create a basic objective methodological approach to apply to other reservoir or lake mapping projects. By incorporating historic geologic maps with sonar and dredge sample validation we were able to objectively identify bottom substrate and provide reservoir users with an accurate map of available benthic habitat. The literature on bottom-typing based on vertical sonar data concentrates on offshore oceanic settings [30] and the Great Lakes [34]. Typically a calibration approach to modeling and mapping is used, where hardness and roughness combinations are assigned sediment types (the “box” method used in [23], with alternatives discussed in [30]). In our study, the ability to resolve bottom sediment types from sonar data alone was limited because there was considerable overlap in the sonar response between sediment classes. Similar difficulties also have been documented for offshore settings [35]. In addition, the sampling methodology has yet to be optimized for reservoir studies. For example, in this study there is a considerable difference between the area represented by the dredge samples and the coverage area of each sonar data point. Mapping could also be enhanced with the addition of sidescan sonar data [36], [37].

From a practical standpoint, it is important to develop robust post-processing techniques based on split- or single-beam echosounders (SBES) due to the fact that the data will have to be interpolated to create a spatially continuous map of benthic habitat, and because these echosounders are a more economical option for fisheries managers. For example, multi-beam systems can cost 2–3 times more than a split-beam system and 10–14 times more than a SBES. Often in acoustic seabed classification studies, most of the cost is associated with data acquisition, leaving little to spend on data processing and interpretation [38]. Our strategy was to pair an inexpensive echosounder and high resolution validation (i.e., ground truthing) with pre-existing supporting spatial data to produce a map of bottom sediments. By including the supporting spatial data (i.e., pre-flood landform and the geologic source materials) we were able to minimize the chance that our interpolation process overlooked discrete features or mischaracterized portions of the benthic habitat. Whereas other data acquisition techniques, such as multibeam systems and sidescan sonar often do not require interpolation due to their wide swath, they are expensive and require computationally intensive data processing [22]. Further, split-beam echosounders can not only aide in benthic habitat studies, but can also be used to calculate fish biomass, due to their divided transducer face that allows the location of targets in three dimensions.

Freshwater lakes and reservoirs offer several advantages for benthic habitat studies when compared to a marine setting, including a discrete study area, a higher probability of existence for supporting data, and easier sediment validation due to shallower depths and smaller average wave heights. Therefore in freshwater lakes and reservoirs the use of a split- or SBES in conjunction with supporting spatial data can be a cost-effective solution for bottom habitat characterization. However, even in a marine setting with multi-beam it would be advantageous to seek out and explore supporting data of any type (e.g., knowledge of marine benthic ecology e.g., [39]; information from Vessel Monitoring Systems e.g., [40]; information on life history traits of species e.g., [41]) as an inexpensive way to corroborate and validate the interpretation of bottom sediments and habitat provided by the interpretation software.

**Figure 8:**
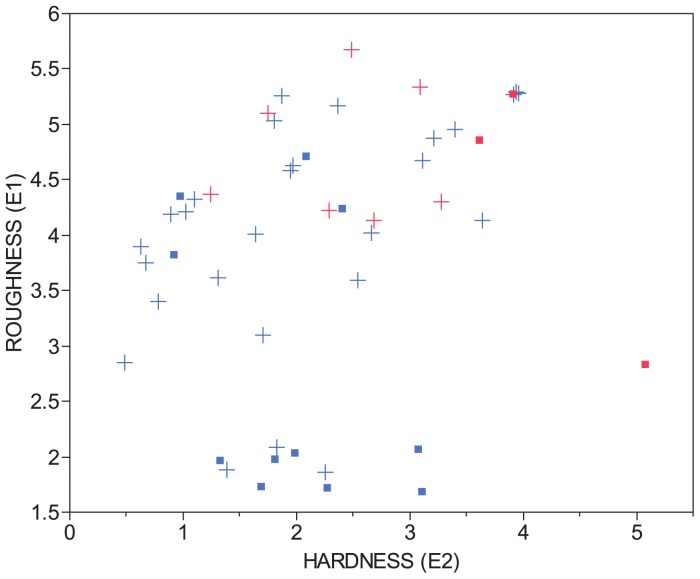
Plot showing roughness versus hardness for muck and organic-rich clay sediments in Hoover Reservoir. Symbols colored blue are organic-rich clays, and those colored red are muck. The symbols are assigned by geologic unit, with squares representing upland units—T, Sh, Mf(a)—and plus symbols representing bottom land units—a, Mf(b).

Of particular interest, in our data, are the occasional large hardness and roughness values (Fig. 8) for soft sediments that should be acoustically soft and smooth. A hypothesis is that these counterintuitive values within the soft sediment classes are caused by increased backscattering from trapped gases sourced from the decomposition of organic matter [42], [43]. This is partially supported by consideration of the distribution of high values between the soft sediment classes and geologic settings (Fig. 8). Firstly, muck has higher organic matter content and therefore is more likely than the organic-rich clays to produce gas. Muck had consistently large hardness and roughness values. In contrast, the organic-rich clay had both high and low roughness values. Within the organic-rich clay unit, a much larger proportion of high roughness values are found in soft bottom areas (geologic units a and Mf(b)) than hard bottom areas. These deep water areas are expected to be more favorable for the accumulation of undisturbed organic deposits than the upland units, which are subjected to wave action and subaerial exposure. Establishing the presence of trapped gases and determining their impact on sonar returns is another potential avenue for the improvement of sonar-based substrate mapping. Especially significant is the potential for temporal instability in sonar results due to changes in gas content and the possibility of using vertical sonar for the monitoring of such changes.

## Conclusion

A methodological framework has been developed to map the bottom sediments in reservoirs and better understand sedimentation patterns. Whereas sonar and dredge sample data are very useful for mapping, interpretation and modeling can be enhanced with a comprehensive exploration of additional predictive data. The configuration of bottom sediments in reservoirs may be related to a wide range of factors, including land cover and geologic properties of the pre-flood landscape, disturbance caused by pre-flood construction and post-flood dredging, and post-flood sedimentary and biological processes. For Hoover Reservoir, we found that pre-flood landform and the geologic source materials were the most useful of the available secondary data. However, each reservoir is a unique system with its own setting, age, history, and range of available data, so data types useful for one cannot be assumed to be applicable to others. Multivariate analysis can provide a better understanding of the range of factors impacting the sonar returns and the reservoir processes most important to predicting the distribution of bottom sediments.

Bottom sediment maps provide insights into the processes and controls on reservoir habitats that are otherwise not readily available. Fisheries managers can use such maps to their advantage. For example, [24] used the maps presented here to determine saugeye *Sander vitreus* X *S. canadense* habitat preferences, which are clearly areas dominated by gravel lag deposits and shale bedrock (Fig. 7). This result led to the management suggestion of stocking saugeye based on available habitat rather than by the surface area of the reservoir, since not all areas provide suitable habitat [24]. Overall, substrate mapping of reservoirs can offer valuable information for managers including tracking habitat changes due to reservoir sedimentation, aiding sport fish stocking decisions based on available habitat, or by directing habitat enhancement efforts.

## Acknowledgments

Stacy Xenakis (ODNR Division of Wildlife) is thanked for providing bathymetry data. Scott Hale (ODNR Division of Wildlife) and Dr. Elizabeth Marschall (The Ohio State University) provided helpful review and guidance for this project.
